# Supplementary material for: Potential Effects of Human Papillomavirus Type Substitution, Superinfection Exclusion and Latency on the Efficacy of the Current L1 Prophylactic Vaccines
Source: Viruses. 2020 Dec 24;13(1):22. doi: 10.3390/v13010022 (PMC7823767; doi:10.3390/v13010022)
Supplement: Supplementary file 1 [file viruses-13-00022-s001.pdf]

Supplementary Table S1. Overview of clinical efficacy studies.

Study Protocol	Location, Study Centre Number, Dates	Study Vaccine No/ Study Arm	Subject Number, Ages	Primary Endpoint	Duration Post-7 Month FU
**P005** Phase IIb	USA (n=16 sites) 1998 - 2004	HPV 16 L1 VLP (40mcg)/ placebo (1193/1198)	N = 2,409 16 -23 year-old women	1. Safety and tolerability of vaccine 2. Efficacy in prevention of persistent HPV 16 infection vs placebo	Mean: 3.1 years Median: 3.9 years
**P007** Phase IIb Dose-ranging study	USA, Europe Latin America (n=23 sites) 2000 - 2004	qHPV VLP vaccine 20/40/40/20mcg 40/40/40/40mcg 80/80/40/80mcg placebo Part A n=52 Part B n=1106	N = 1,158 16 -23 year-old women	Part A: General tolerability Part B: 1. Identify formulations with acceptable type specific anti- HPV responses 2. Efficacy in prevention of persistent HPV 6,11, 16, 18 infection and clinical disease cf placebo 3. General tolerability	Mean: 2.4 years Median: 3.0 years Prot. 7-10 Mean: 4.5 years Median: 4.9 years
**P013** Phase III FUTURE I	North America, Latin America, Europe, Asia-Pacific (n=62 sites) 2001 - 2005	qHPV VLP vaccine 20/40/40/20mcg/ Placebo (2717 / 2725)	N=5,455 16- 23 year-old women	Co-primary endpoints: i) External genital lesion: efficacy in reducing HPV 6,11,16,18-related EGL (=genital warts, VIN, VaIN, vulvar or vaginal cancer) cf placebo ii) Cervical endpoint: efficacy in reducing the incidence of HPV 6,11, 16,18-related CIN (any grade), AIS or cervical cancer cf placebo - Safety and tolerability	Mean: 1.7 years Median: 2.4 years Updated Mean: 2.4 years Median: 2.9 years
**P015** Phase III FUTURE II	North America, Latin America, Europe, Asia-Pacific (n=90 sites) 2002 - 2005	qHPV VLP vaccine 20/40/40/20mcg/ Placebo (6082 / 6075)	N=12,167 16- 26 year-old women	Primary Cervical endpoint: efficacy in reducing the incidence of HPV 6,11,16,18-related CIN 2/3, AIS or invasive cervical cancer in HPV naïve subjects	Mean: 1,4 years Median: 2.0 years Updated Mean: 2.4 years Median: 2.9 years

https://www.ema.europa.eu/en/documents/variation-report/gardasil-h-c-703-ii-13-epar- assessment-report-variation_en.pdf (p.5)

Supplementary Figure S1: Study Populations

Per-protocol efficacy (PPE) population:

- Received all 3 doses of study vaccine
- Were seronegative to relevant vaccine HPV type(s) at Day 1
- Were PCR negative to relevant vaccine HPV type at Day 1 and at Month 7
- Did not deviate from the protocol
- Cases counted starting 30 days post-dose 3 (Month 7)

The Per-protocol efficacy (PPE) population was used as the primary analysis population for efficacy and includes subjects naïve to relevant vaccine HPV types.

Modified intention to treat (MITT) populations :

- MITT-2 population
- Restricted MITT-2 (R-MITT2)
- MITT-3 population: general population (ITT)

Two MITT populations were used for the analysis of efficacy with respect to the population benefit endpoints (i.e., evaluation of the impact of Gardasil on the incidence of disease caused by vaccine or non-vaccine HPV types). Importantly, these populations differ from those in the marketing authorisation application with respect to the extent of HPV type testing. This updated report, include additional testing for 10 common non-vaccine HPV types (HPV 31, 33, 35, 39, 45, 51, 52, 56, 58 and 59 in all swabs and tissue specimens obtained during the phase III studies.

MITT-2 population

- Received at least one dose of study vaccine
- Were seronegative and PCR-negative to all 4 vaccine HPV types at Day 1
- Were PCR-negative at Day 1 to the appropriate non-vaccine HPV type (31, 33, 35, 39, 45, 51, 52, 56, 58, or 59) or had a negative Day 1 Pap test result
- Had at least one follow-up visit after 1 month following the first injection
- Cases were counted starting after Day 30

This population represents virginal HPV naïve young women but who could be infected with HPV before or after the completion of the vaccination period.

Restricted MITT-2 (R-MITT2)

- Received at least one dose of study vaccine
- Were seronegative and PCR negative to all 4 vaccine HPV types at Day 1
- Were PCR negative to HPV 31, 33, 35, 39, 45, 51, 52, 56, 58, and 59 at Day 1 and had a negative Pap test result at Day 1
- Had at least one follow-up visit after 1 month following the first injection
- Cases were counted starting after Day 30

The RMITT-2 was designed to approximate a population of adolescent and young adult women who were either sexually-naïve or sexually-experienced and had not yet been exposed to *any* HPV type but who could be infected with HPV before or after the completion of the vaccination period.

MITT-3 population: general population (ITT)

- Received at least one dose of study vaccine

- Regardless of PCR status at Day 1
- Cases counted starting 30 days after Day 1

Compared with the main analysis populations, the MITT-3 population also includes subjects who were already infected with a vaccine or non-vaccine HPV type at Day 1 and subjects who had evidence of CIN at Day 1 (e.g., an abnormal Pap test at Day 1). Such a population approximates the general population of sexually- active 16- to 26-year-old girls and provides a real world estimate of efficacy in the vaccinated population.
